# Supplementary material for: Newly Synthesized Oxygenated Xanthones as Potential P-Glycoprotein Activators: In Vitro, Ex Vivo, and In Silico Studies
Source: Molecules. 2019 Feb 15;24(4):707. doi: 10.3390/molecules24040707 (PMC6412186; doi:10.3390/molecules24040707)
Supplement: Supplementary file 1 [file molecules-24-00707-s001.pdf]

molecules

SUPPLEMENTARY MATERIALS

*Article*

# Newly synthetized oxygenated xanthones as potential P-glycoprotein activators—*in vitro*, *ex vivo* and *in silico* studies

**Eva Martins[1], Vera Silva[1], Agostinho Lemos[2], Andreia Palmeira[2], Ploenthip Puthongking[3], Emília Sousa[2,4]*, Carolina Rocha-Pereira[1], Carolina I. Ghanem[5], Helena Carmo[1], Fernando Remião[1]* and Renata Silva[1]***

[1] UCIBIO-REQUIMTE, Laboratório de Toxicologia, Departamento de Ciências Biológicas, Faculdade de Farmácia, Universidade do Porto, Rua de Jorge Viterbo Ferreira, 228, 4050-313 Porto, Portugal; evagilmartins18@gmail.com (E.M.); veralssilva17@gmail.com (V.S.); mcamorim@ff.up.pt (C.R-P.); helenacarmo@ff.up.pt (H.C.); remiao@ff.up.pt (F.R.); rsilva@ff.up.pt (R.S.)

[2] Laboratório de Química Orgânica e Farmacêutica, Departamento de Ciências Químicas, Faculdade de Farmácia, Universidade do Porto, Rua Jorge Viterbo Ferreira 228, 4050-313 Porto, Portugal; up201002662@ff.up.pt (A.L.); apalmeira@ff.up.pt (A.P.); esousa@ff.up.pt (E.S.)

[3] Faculty of Pharmaceutical Sciences, Khon Kaen University, 40002 Khon Kaen, Thailand; pploenthip@gmail.com

[4] Centro Interdisciplinar de Investigação Marinha e Ambiental (CIIMAR/CIMAR), Universidade do Porto, Rua dos Bragas 289, 4050-123 Porto, Portugal

[5] Universidad de Buenos Aires, CONICET, Facultad de Farmacia y Bioquímica, Instituto de Investigaciones Farmacológicas (ININFA), Buenos Aires, Argentina; cghanem@ffyb.uba.ar

* Correspondence: esousa@ff.up.pt (E.S.), remiao@ff.up.pt (F.R.), rsilva@ff.up.pt (R.S.); Tel.: +351-22-0428598 (F.R. and R.S.); +351-22-0428689 (E.S.)

*Correspondence: remiao@ff.up.pt, esousa@ff.up.pt, rsilva@ff.up.pt; Tel.: +351-22-042889

Academic Editor: name
Received: date; Accepted: date; Published: date

molecules

**Figure S1**

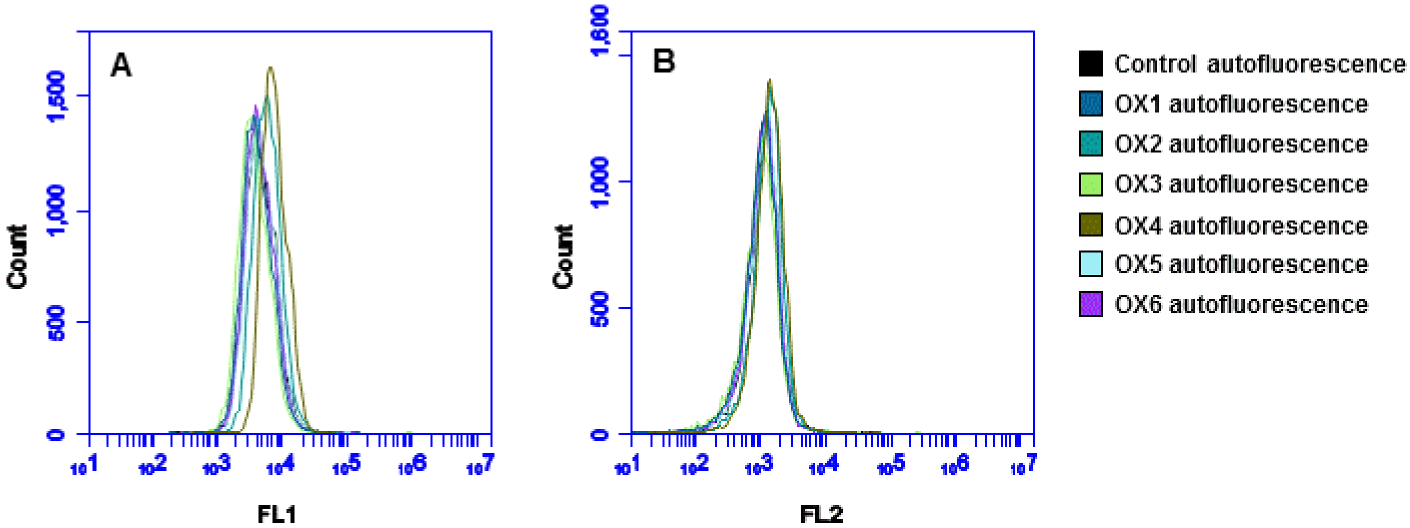


**Figure S1.** Representative histograms of flow cytometry analysis of Caco-2 cells autofluorescence in the 530 ± 15 nm band-pass filter (A - FL1 detector) and in the 585 ± 40 nm band-pass filter (B - FL2 detector), 24 h after the incubation with the tested oxygenated xanthones OX 1-6 (20.0 µM).

**Figure S2**

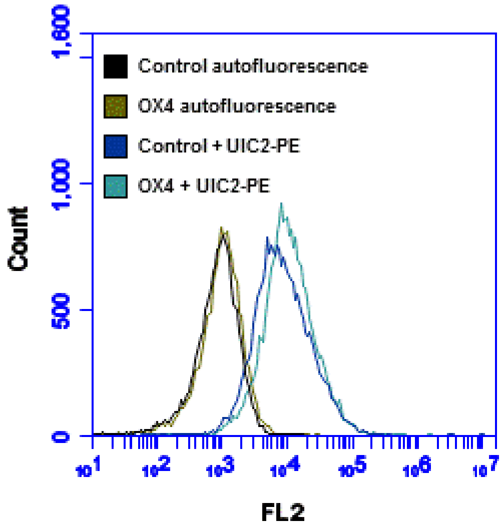


**Figure S2.** Representative histograms of flow cytometry analysis of P-glycoprotein (P-gp) expression, evaluated 24 h after exposure of Caco-2 cells to the oxygenated xanthone OX4 (20.0 µM), using the UIC2-PE monoclonal antibody (585 ± 40 nm band-pass filter - FL2 detector).

**Figure S3**

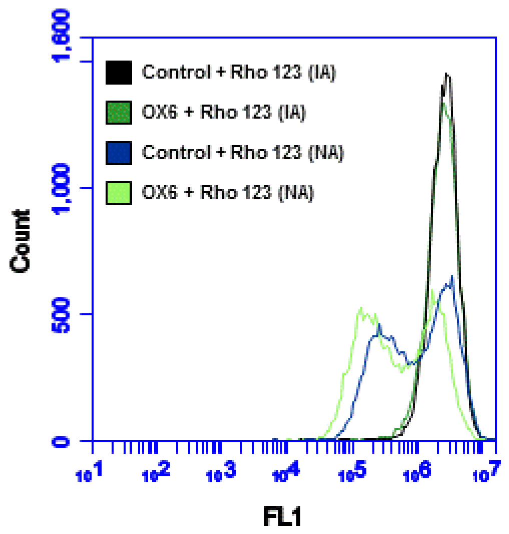


**Figure S3.** Representative histograms of flow cytometry analysis of P-glycoprotein (P-gp) activity, evaluated 1 h after exposure of Caco-2 cells to the oxygenated xanthone OX6 (20.0 µM), using Rhodamine (Rho 123) as a fluorescent substrate (530 ± 15 nm band-pass filter - FL1 detector). IA (inhibited accumulation), NA (normal accumulation).

**Figure S4**

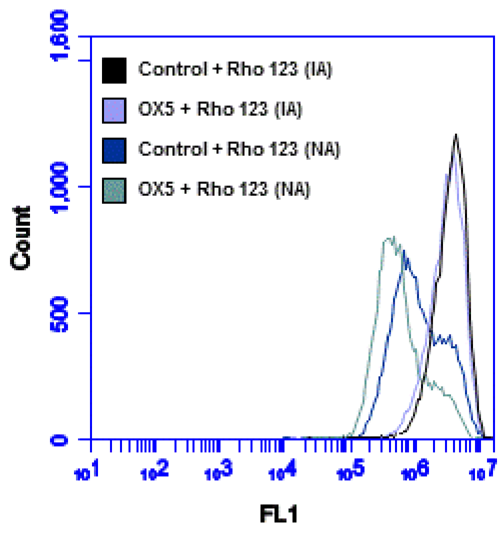


**Figure S4.** Representative histograms of flow cytometry analysis of P-glycoprotein (P-gp) activity, evaluated 24 h after exposure of Caco-2 cells to the oxygenated xanthone OX5 (20.0 µM), using Rhodamine (Rho 123) as a fluorescent substrate (530 ± 15 nm band-pass filter - FL1 detector). IA (inhibited accumulation), NA (normal accumulation).

**Figure S5**

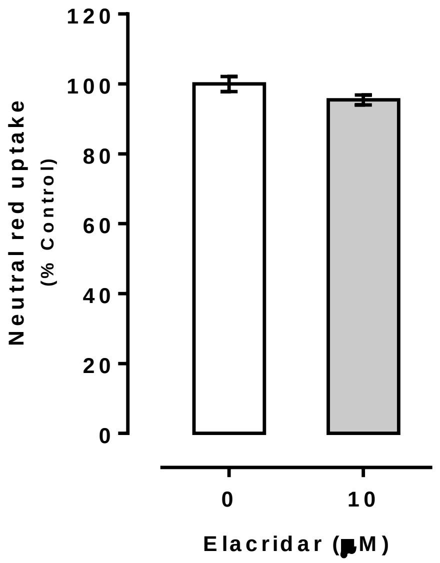


**Figure S5.** Elacridar (Ela, 0 - 10.0 μM) cytotoxicity in Caco-2 cells evaluated by the Neutral Red (NR) uptake assay, 24 h after incubation. Results are presented as mean ± SEM from 3 independent experiments, performed in triplicate. Statistical comparisons were made using the Unpaired *t* test.

**Figure S6**

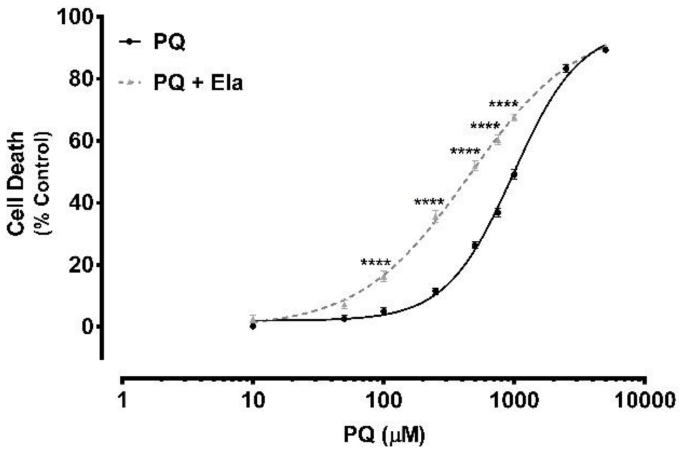


**Figure S6.** Paraquat (PQ) concentration-response (cell death) curve obtained in the absence (PQ) or in the presence of 10.0 μM Elacridar (PQ + Ela). Results are presented as mean ± SEM from 4 independent experiments (performed in triplicate). Concentration-response curve was fitted using least squares as the fitting method and the comparison between PQ and PQ + Ela curves (LOG $EC_{50}$, TOP, BOTTOM, and Hill Slope) was made using the extra sum-of-squares F test. Statistical comparisons were made using Two-way ANOVA, followed by the Sidak's multiple comparisons post hoc test (****$p < 0.0001$ PQ + Ela vs. PQ). In all cases, p values < 0.05 were considered statistically significant.

## Table S1

**Table S1.** $EC_{50}$ (half-maximum-effect concentrations), TOP (maximal effect), BOTTOM (baseline) and Hill Slope values of the paraquat (PQ) concentration-response curve, with (PQ + Ela) or without (PQ) simultaneous exposure to Elacridar (10.0 µM).

| | PQ | PQ + Ela |
|---|---|---|
| **$EC_{50}$** (half-maximum-effect concentrations, µM) | **982.4** | **450.3******** |
| **Top** (maximal cell death, % control) | 96.65 | 97.76 |
| **Bottom** (baseline, % control) | 2.008 | -0.2621 |
| **Hill slope** | **1.694** | **1.039******** |
| **Curve *p* value** (Comparison between the fitted curves) | - | **< 0.0001** |

Concentration-response curves were fitted using least squares as the fitting method and the comparisons between PQ and PQ + Ela curves were made using extra sum-of-squares F test. In all cases, p values < 0.05 were considered significant (****p < 0.0001 for PQ vs. PQ + Ela). Bold is used when significant exists.
